# Comparative analysis of a BAC contig of porcine chromosome 13q31-q32 and human chromosome 3q21-q22

**DOI:** 10.1186/1471-2164-6-133

**Published:** 2005-09-21

**Authors:** Mario Van Poucke, David Bourry, François Piumi, Marc Mattheeuws, Alex Van Zeveren, Patrick Chardon, Luc J Peelman

**Affiliations:** 1 Department of Animal Genetics and Breeding, Faculty of Veterinary Medicine, Ghent University, Heidestraat 19, B-9820 Merelbeke, Belgium; 2 Department of Organic Chemistry, Faculty of Sciences, Ghent University, Krijgslaan 281 S4, B-9000 Ghent, Belgium; 3 Laboratoire de Radiobiologie et d'Etude du Génome, UMR INRA-CEA, F-78352 Jouy en Josas cedex, France

## Abstract

**Background:**

The gene(s) encoding the ETEC F4ab/ac receptors, involved in neonatal diarrhoea in pigs (a disease not yet described in humans), is located close to the TF locus on Sscr13. In order to reveal and characterize possible candidate genes encoding these receptors, a porcine physical map of the TF region is indispensable.

**Results:**

A contig of 33 BAC clones, covering approximately 1.35 Mb surrounding the TF locus on Sscr13q31-q32, was built by chromosome walking. A total of 22,552 bp from the BAC contig were sequenced and compared with database sequences to identify genes, ESTs and repeat sequences, and to anchor the contig to the syntenic region of the human genome sequence (Hsap3q21-q22). The contig was further annotated based on this human/porcine comparative map, and was also anchored to the Sanger porcine framework map and the integrated map of Sscr13 by RH mapping.

**Conclusion:**

The annotated contig, containing 10 genes and 2 ESTs, showed a complete conservation of linkage (gene order and orientation) with the human genome sequence, based on 46 anchor points. This underlines the importance of the human/porcine comparative map for the identification of porcine genes associated with genetic defects and economically important traits, and for assembly of the porcine genome sequence.

## Background

Neonatal diarrhoea, often caused by ETEC F4 bacteria, is a common problem in pig production. These bacteria use their fimbriae to adhere to specific receptors on the brush borders of enterocytes of their host. This adhesion is a prerequisite for infection and promotes bacterial colonization of the small intestine. The colonizing bacteria produce enterotoxins that stimulate the secretion of water and electrolytes into the lumen of the small intestine and lead to diarrhoea and often death in neonatal pigs [1]. ETEC F4 resistance, acquired by receptor phenotype differences of the host, seems to be inherited as an autosomal recessive Mendelian trait [2], whereby the gene(s) encoding the ETEC F4ab/ac receptors have been linked to several loci on Sscr13 [3-7]. Based on the tight linkage of the ETEC F4ab/ac receptor loci to microsatellite markers Swr926 (Locus P) and Swc22 (Locus G) by Peelman [5], a BAC contig covering this region and containing TF was built by chromosome walking. The contig was annotated by comparing BAC sequences with sequences from nucleotide databases and by comparative mapping with the human genome sequence in order to provide a basis for the identification of the ETEC F4ab/ac gene(s) by the candidate gene approach.

## Results and discussion

### Construction of the BAC contig

**Figure 1:**
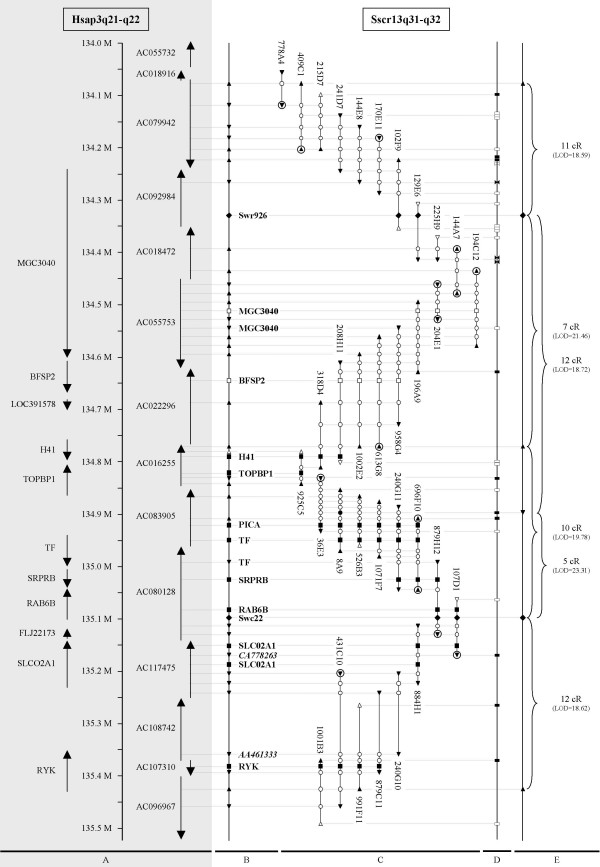
**Comparative map of the annotated BAC contig with its syntenic region on Hsap3q21-q22**. The contig is drawn in part C. Black triangles represent BAC end sequences from which primers were designed to construct the contig. The black circle represents the only internal BAC sequence from which primers were designed to construct the contig. White circles show overlaps of these BAC sequences with other BAC clones. White triangles represent BESs from which it was impossible to design primers. The triangles point towards the 3'-side of the BAC clone. Encircled triangles represent BAC ends that are also present in the Sanger framework map. Black diamonds represent microsatellite positions. Black squares represent genes annotated by PCR, whereas white squares represent genes annotated by hybridization. Annotated sequences (genes are in regular, ESTs in italic) from the BAC contig are represented on a plane map (B) and their homology with the human genome sequence is illustrated with dotted lines (A). The orientation of the human genes and finished HTGs (used to assemble the human genome sequence) are represented in (A) by arrows [10]. Repeat sequences (white rectangle = LINE, black rectangle = SINE, black bow = LTR element, white bow = DNA element) are shown on a plane map in D. The RH mapping results are shown in E.

The construction of the BAC contig was started at 2 microsatellite marker loci, Swr926 and Swc22, estimated to be 1 cM apart from each other and closely linked to the ETEC F4ab/ac receptor loci, according to the porcine genetic map of Peelman [5]. From those 2 loci, 2 subcontigs were built by chromosome walking in both directions until the gap between the 2 was filled. The resulting BAC contig, comprising 33 BAC clones, is shown in Figure 1C All 66 BAC ends were sequenced and submitted to the GenBank database as GSSs [GenBank:CG993013-CG993078]. On 4 occasions, 2 BAC clones turned out to possess the same end (5'-215D7 with 5'-409C1, 129E6-3' with 225H9-3', 5'-613G8 with 5'-1002E2, and 5'-696F10 with 240G11-3'). From 52 of the 62 unique sequences, primers were designed to construct the contig and to screen for new overlapping clones. By dividing the total number of overlaps between the BAC ends and the BAC clones by the total number of BAC ends an estimated contig depth of 3.3 was calculated. Since the average length of the BAC inserts is 135,000 bp, we have covered a region of approximately (33/3.3) × 135,000 = 1.35 Mb.

### Annotation of the BAC end sequences

A total of 22,552 bp of the BAC contig (62 unique BAC ends and 1 internal BAC fragment [GenBank:CZ692943]) were sequenced and annotated by NIX [8]. The sequences had an overall GC content of 41.48%, which is less than the 46.17% for Sscr7q found in an analogous study of Barbosa and co-workers [9].

The BESs contained 2 gene fragments (MGC3040 and TF) and 2 ESTs (CA778263 and AA461333) located on the human genome (Figure 1A–C) [10]. In 35 of the 62 BESs, homologous sequences could be found within 12 consecutive finished HTGs used to assemble the Hsap3q21-q22 region of the human genome sequence (Figure 1A–C) [10]. These homologies were studied in detail by BLAST 2 sequence comparisons of the BESs with their orthologs (based on the 35.1 latest human genome build). Repeat sequences were excluded (RepeatMasker) and only single hits were taken into account. Orthologous sequences longer than 50 bp had on average a length of 150 bp, a sequence identity of 80% and an e-value of 1e-20. Smaller fragments were only considered as orthologs if at least 2 of them were located close to each other at their expected orthologous position. An extended conservation of synteny between Sscr13 and Hsap3 was already shown by the comparative map of Van Poucke and co-workers [11], based on chromosome painting results of Goureau and co-workers [12]. But taking into account the orientation of the finished HTGs and the position of the orthologous sequences within these HTGs, a perfect comparative map could be established showing even 100% conserved linkage in this region.

Based on this comparative map the BAC contig covers approximately 1.40 Mb of the human genome (from 134.075 Mb to 135.475 Mb on Hsap3) [10], which is close to the BAC contig length calculated above. The BAC sequences also contained 17 LINEs, 10 SINEs, 3 LTR elements and 1 DNA element (Figure 1D), resulting in an average density of 0.77 LINEs/kb, 0.45 SINEs/kb, 0.14 LTR elements/kb and 0.05 DNA elements/kb. Barbosa and co-workers [9] found an average density of 0.35 LINEs/kb, 0.61 SINEs/kb and 0.17 LTR+DNA elements/kb on Sscr7q.

### Comparative mapping with the human syntenic region

Based on this detailed comparative map between Hsap3q21-q22 and the BAC contig, the latter could be annotated by comparative mapping. H41, TOPBP1, TF, SRPRB, RAB6B, SLCO2A1 and RYK could be found in the contig by PCR (Figure 1A–C). Also PICA, a gene not yet described in human but located on the pig EST map of the NCBI human genome map viewer [10] between TOPBP1 and TF, was found in the contig by PCR at the orthologous region (Figure 1A–C). It showed sequence homology with the finished HTG sequence AC083905. MGC3040 and BFSP2 could be found in the contig by BAC colony hybridisation (Figure 1A–C). For MGC3040, TF and SLCO2A1 two regions of the gene were annotated in the contig. Their locations showed that those genes were organised in the same orientation as in human. All the comparative mapping results confirmed the conserved linkage (gene order and orientation) based on the sequence homologies of the BESs (Figure 1A–C).

Fifteen BAC ends are also located on the Sanger porcine framework map [13]. On average, they show 98.5% sequence identity, and are located in the same order (Figure 1C). This was expected because (1) the framework map was constructed by fingerprinting and BES alignment on the human sequence, and (2) this region shows 100% conserved linkage with the human genome. So, for this region, the Sanger map assembly, based on the assumption of conservation between both species, is correct. But because of inter- and intrachromosomal rearrangements between the human and the porcine chromosomes [11,12], the Sanger framework map contains some errors. This underlines the importance of the chromosome walking approach for the development of an exact map.

Based on the characteristics of the genes annotated in this contig, SLCO2A1 could be a candidate gene encoding the ETEC F4ab/ac receptor. It is a single copy gene encoding the prostaglandin transporter, a 12-transmembrane organic anion cell surface transporter that is expressed in the small intestine. The presence of different mRNA transcripts suggests that several functionally distinct mRNAs may arise by alternative splicing and/or alternative promotors [14]. It is also assumed that SLCO2A1 contains several different substrate binding sites, to which binding does not always result in substrate translocation across the membrane [15].

### RH mapping

During chromosome walking, 4 loci were mapped with the IMpRH panel (data are submitted to the IMpRH server [16]) in order to detect possible chromosome jumping, to estimate the remaining gap between the 2 subcontigs, and to anchor the contig to the integrated comparative map [11]. Using the IMpRH server, 2-point distances were calculated between BAC ends 409C1-3', 5'-613G8, 5'-991F11 and an internal sequence of BAC 8A9, and microsatellite markers Swr926 and Swc22, that were previously mapped on the IMpRH map (Figure 1E) [17]. Based on these distances, the contig covers a region of approximately 40 cR. Thus, 1 cR equals approximately 33.750 kb in our contig. The distance between Swr926 and Swc22 was measured as 17 cR. Because the same distance was measured as 1 cM on a linkage map of Peelman [5], the cR/cM ratio in our contig is 17. Hawken and co-workers [17] measured values for Sscr13 of 59.9 kb/cR and 30.4 cR/cM with the linkage map of Rohrer and co-workers [18].

## Methods

### Primer design and amplicon verification

All primers, designed with Primer3 [20], were confirmed to not generate an amplicon of the same length with bacterial DNA as a template. Primers used for RH mapping were also checked not to generate an amplicon of the same length with hamster DNA as a template. The construction of the BAC contig was started with primers amplifying porcine microsatellite markers Swr926 [GenBank:AF235467] and Swc22 [GenBank:AF225193]. During the construction of the BAC contig, new primers were designed based on the BESs [GenBank:CG993013-CG993078]. Information on those primers can be found in the corresponding GenBank files. For annotation by comparative mapping with the human genome, primers were designed based on orthologous human and/or porcine sequences. Information on new primers is presented in Table 1. These PCR products were cloned in pCRII (Invitrogen, Merelbeke, Belgium), sequenced for verification with the Thermo Sequenase Primer Cycle Sequencing Kit (Amersham Biosciences, Uppsala, Sweden) and submitted to GenBank [GenBank:AY5182650-AY5182658, DQ104835, DQ104841]. Because of sequence homology, primers for PICA were confirmed to not amplify TF. Primers for RYK were described earlier [11].

**Table 1:** Information on new primers for genes annotated by PCR

Gene	Forward Primer (5'- 3')	Annealing temperature
Porcine Acc.No.	Reverse Primer (5'- 3')	Amplicon size
H41	GGCAAGAGTGAAGCAAATGG	60°C
AY518265	TCAAAAACATAACCCCAGCAA	395 bp
TOPBP1	CCTGAATCTCTTTATCCACATACTT	57°C
AY518266	CATTTGATGGTGCTGACTCTT	318 bp
PICA	TGGACGCGAAGCTCTAT	59°C
U36916	TCCGAGTTACAATTCAAGATG	1.286 bp
TF (exon 2)	CCAATAAGTGCTCCAGTTTC	56°C
X12386	CCCTGATGGCTTTGATG	111 bp
SRPRB	CGCCTTCCATCCCTACCT	58°C
AY518267	AACCGCCCTTTGACTGCT	756 bp
RAB6B	CATTGGGATTGACTTCTTGTC	58°C
AY518268	GATGTAGCTGGGGATCAGG	313 bp
SLCO2A1 (exon 3)	GCCGTCCTCATCATCTTTGT	60°C
DQ104835	GAAGTGCGGGAGGGTGA	117 bp
SLCO2A1 (exon 9)	CCTTGGGGATGCTGTTTG	60°C
DQ104841	TGGAGATGGTGATGATGGTG	96 bp

### BAC screening and contig building by chromosome walking

The INRA porcine BAC library was screened by PCR [21]. Approximately 20 μg BAC DNA was purified from a 100 ml culture of the isolated BAC clones by using the Qiagen Plasmid Midi Kit (Westburg, Leusden, The Netherlands). The primers used to isolate the BAC clones were used to amplify the same amplicon on 20 ng BAC DNA for verification. Both ends of the isolated BAC clones were sequenced with 5 μg BAC DNA as template by using the Thermo Sequenase Primer Cycle Sequencing Kit (Amersham Biosciences, Uppsala, Sweden). Primers based on those BESs were used to construct the contig by defining overlaps with all other BAC clones. Primers at both ends of the growing subcontigs were used to screen the BAC library for new overlapping clones until the gap between Swr926 and Swc22 was filled.

### Annotation

Annotation of the contig was performed by analyzing all BAC sequences on the NIX server (allowing integration and display of many gene identification programs, such as BLAST against EMBL, EST, STS and GSS databases [8], but not operational anymore), and by comparative mapping using PCR and BAC colony hybridization. These and similar sequence comparisons such as BLAST 2, can also be performed via the NCBI BLAST server [22]. Gene symbols, names and positions were based on the NCBI Gene Entrez [23] and NCBI Map viewer [10] with the latter also used for the identification of the human HTGs.

### BAC colony hybridization

For annotation purposes by comparative mapping with the human genome, 2 IMAGE clones (3163990 [GenBank:BC000568] at the MGC3040 locus and 2472940 [GenBank:AI954686] at the BFSP2 locus), located in the human syntenic region, were ordered (MRC geneservice, Cambridge, UK). Inserts of these clones were used as radiolabeled probes for BAC colony hybridization.

### RH mapping

During chromosome walking, 4 loci were mapped on the IMpRH panel [24] in order to detect possible chromosome jumping, to estimate the remaining gap between the 2 subcontigs, and to anchor the contig to the integrated comparative map [11]. Swr926 and Swc22 [17] and TF [25] were already located on the IMpRH map.

## Conclusion

A porcine BAC contig containing 33 BAC clones and covering approximately 1.35 Mb of Sscr13q31-q32 was constructed. The annotated contig, containing 10 genes and 2 ESTs, showed a complete conservation of linkage with Hsap3q21-q22, based on 46 anchor points, providing further evidence for conservation of linkage on a fine scale. This underlines the importance of the comparative mapping strategy between human and pig, not only in the search for genes in pig but also as a basis for the assembly of the porcine genome [13,19]. The contig also contains 15 anchor points with the Sanger porcine framework map [13], 4 anchor points (Swr926, Swc22, TF and RYK) with the integrated map of Sscr13 [11] and 2 (Swr926, Swc22) with the porcine Map Viewer [10].

## List of abbreviations

BAC bacterial artificial chromosome

BES BAC end sequence

BFSP2 beaded filament structural protein 2, phakinin

bp basepairs

cM centiMorgan

cR centiRay

EMBL European Molecular Biology Laboratory

EST expressed sequence tag

ETEC enterotoxigenic *Escherichia coli*

GSS genomic survey sequence

H41 hypothetical protein H41

Hsap *Homo sapiens*

HTGs high throughput genomic sequences

IMpRH INRA-University of Minnesota porcine Radiation Hybrid

kb kilobasepairs

LINE long interspersed nuclear elements

LTR long terminal repeat

Mb megabasepairs

MGC3040 hypothetical protein MGC3040

PICA porcine inhibitor of carbonic anhydrase

RAB6B RAB6B, member RAS oncogene family

RH radiation hybrid

RYK RYK receptor-like tyrosine kinase

SINE short interspersed nuclear elements

SLCO2A1 solute carrier organic anion transporter family, member 2A1

SRPRB signal recognition particle receptor, B subunit

Sscr *Sus scrofa*

STS Sequence Tag Site

TF transferrin

TOPBP1 topoisomerase (DNA) II binding protein 1

## Authors' contributions

MVP coordinated this work, carried out the contig building and the primer design, and drafted this manuscript. DB carried out the contig annotation. FP assisted the BAC screening. MM carried out the sequencing. AVZ and LJP designed the project. PC supervised the BAC screening. All authors read and approved the final manuscript.
